# Emerging infectious diseases in an island ecosystem: the New Zealand perspective.

**DOI:** 10.3201/eid0705.017501

**Published:** 2001

**Authors:** J A Crump, D R Murdoch, M G Baker

**Affiliations:** Division of Bacterial and Mycotic Diseases, National Center for Infectious Diseases, Centers for Disease Control and Prevention, Atlanta, Georgia 30333, USA. znc0@cdc.gov

## Abstract

Several unique features characterize infectious disease epidemiology in New Zealand. Historically, well-organized, government-run control programs have eliminated several zoonoses. More recently, however, communicable disease control has been mixed. Rates of rheumatic fever, tuberculosis, and enteric infectious are high, and rates of meningococcal disease are increasing. These diseases are over-represented in New Zealanders of Polynesian descent, who generally live in more deprived and overcrowded conditions than do those of European descent. Measles and pertussis epidemics are recurring because of inadequate vaccine coverage, despite a well-developed childhood immunization program. A progressive response to the HIV epidemic has resulted in relatively low rates of infection, particularly among injecting drug users; however, the response to other sexually transmitted infections has been poor. A key challenge for the future is to build on successful strategies and apply them to persisting and emerging infectious disease threats in a small, geographically isolated country with limited economic resources.


### Emerging Infectious Diseases in an Island Ecosystem: The New Zealand Perspective

**John A. Crump,* David R. Murdoch,† and Michael G. Baker‡**
*Centers for Disease Control and Prevention, Atlanta, Georgia, USA; †Canterbury Health Laboratories, Christchurch, New Zealand; and ‡Institute of Environmental Science and Research, Porirua, New Zealand

Several unique features characterize infectious disease epidemiology in New Zealand. Historically, well-organized, government-run control programs have eliminated several zoonoses. More recently, however, communicable disease control has been mixed. Rates of rheumatic fever, tuberculosis, and enteric infections are high, and rates of meningococcal disease are increasing. These diseases are overrepresented in New Zealanders of Polynesian descent, who generally live in more deprived and overcrowded conditions than do those of European descent. Measles and pertussis epidemics are recurring because of inadequate vaccine coverage, despite a well-developed childhood immunization program. A progressive response to the HIV epidemic has resulted in relatively low rates of infection, particularly among injecting drug users; however, the response to other sexually transmitted infections has been poor. A key challenge for the future is to build on successful strategies and apply them to persisting and emerging infectious disease threats in a small, geographically isolated country with limited economic resources.

### Unique Historical and Epidemiologic Features

New Zealand (known as Aotearoa in Maori), a South Pacific nation with a population of 3.8 million, is the largest island group in Polynesia (Figure). It shares strong biologic similarities with other islands in Polynesia, although it is often wrongly grouped with Australia. New Zealand has several unique features of special interest in the study of emerging infectious diseases. These include unusual native fauna, lack of native terrestrial mammals, and recent incursions of exotic fauna. With exotic fauna came a limited range of zoonoses that were successfully controlled and excluded by a strict quarantine system. Furthermore, New Zealand has unusually high rates of some endemic infectious diseases and delayed impact from infectious diseases emerging in other parts of the globe.

New Zealand developed from the margin of the southern landmass of Gondwana. Separation from Australia and Antarctica occurred 100 to 75 million years ago (1). The country is one of the most geographically isolated and remote temperate islands in the world. Until recently, this isolation allowed a peculiar native fauna to evolve in the absence of natural predators and incursions by exotic species. The only native mammals of New Zealand are two genera of bats (*Chalinolobus* spp. and *Mystacina* spp.). Native bird and insect species evolved to fill ecologic niches that in other countries are occupied by mammalian species. Native parasitic arthropods matched the limited range of terrestrial fauna. It is thought that relatively few microorganisms capable of infecting humans existed in New Zealand before the arrival of the first settlers.

The first evidence of humans in New Zealand dates to approximately 700 years ago. The colonizing Polynesian population is now known as the Maori. Strong oral and artistic traditions maintained by the Maori are not particularly revealing of early health history.

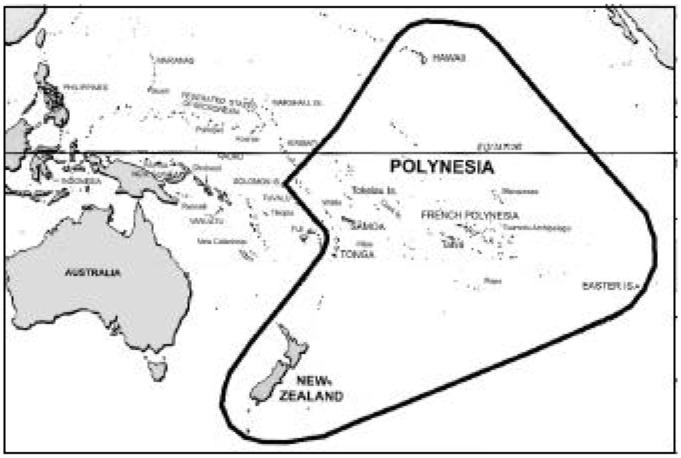

Figure. New Zealand and the region of Polynesia.

Address for correspondence: John A. Crump, Division of Bacterial and Mycotic Diseases, National Center for Infectious Diseases, Mailstop A38, Centers for Disease Control and Prevention, 1600 Clifton Road, Atlanta, GA 30333, USA; fax: 404-639-2205; e-mail: zcn0@cdc.gov

European exploration began in 1642 and continued until 1769. Introduced epidemic disease occurred among the Maori population from the 1790s. The introduction of infectious agents such as *Influenza virus* (2) parallels similar introductions elsewhere in the world. Colonization, primarily from Great Britain, followed in the 1790s. Migrants brought a range of infectious diseases endemic in Europe.

New Zealand's native fauna does not include recognized intermediate hosts for human pathogens. The introduction of exotic terrestrial mammals created a new potential for zoonotic disease. In the 14th century, early Polynesian immigrants introduced the Polynesian rat (*Rattus exulans*) and, much later, the dog. However, the arrival of Europeans with a vast range of exotic species 200 years ago brought about one of the most massive recent introductions of new species into a virgin environment.

British explorer Captain James Cook introduced pigs (*Sus scrofa*) and goats (*Capra hircus*) to New Zealand in 1773. The brown rat (*R. norvegicus*), the black rat (*R. rattus*), and the house mouse (*Mus musculus*) were introduced 150 to 200 years ago. Various animals followed, including hedgehogs (*Erinaceus europaeus*), three species of the genus *Mustela* (stoat, weasel, and ferret), various species of deer (including *Dama dama*), chamois (*Rupicapra rupicapra*), thar (*Hemitragus jemlahicus*), hares (*Lepus europaeus*), rabbits (*Oryctolagus cuniculus*), wallabies (*Macropus* sp.), and the Australian brush-tailed possum (*Trichosurus vulpecula*). In addition, domestic animals, including cats, cattle, sheep, and horses, were introduced (3,4).

Along with exotic fauna came a variety of ectoparasites, some of which were potential vectors for arthropod-borne disease. These included the dog flea (*Ctencephalides canis*), the cat flea (*C. felis felis*), the human body louse (*Pediculus humanus*) and various animal louse species, several culicine mosquito species (including *Aedes notoscriptus, A. australis*, and *Culex quinquefasciatus*), a limited number of tick species (including *Ixodes holocyclus*), and the oriental rat flea (*Xenopsylla cheopis*) (5).

Several factors limited the range of zoonoses introduced to New Zealand. These included a small number of animal sources (almost exclusively from the British Isles, Australia, and Chile) and the selection of only healthy stock for transportation. Although settlement and trade often came via Australia, the extended sea voyage to New Zealand during the early European period provided a form of enforced quarantine for some diseases (3,6). Only diseases that could persistently circulate in the crowded conditions of these voyages and those that persisted in a chronic state in humans or animals were imported. The opportunity to take advantage of an island free of most mammalian diseases was recognized by agriculturalists and government, and strict quarantine practices were rapidly put in place. Since primary production has been the main contributor to New Zealand's economy, there is a strong interest in preventing the importation of animal diseases, many of which are potential zoonoses. New Zealand maintains one of the most strict quarantine systems in the world.

This system has been highly successful but has not been immune to biocriminal acts, such as the illegal importation of *Rabbit hemorrhagic disease virus*, probably from Australia, first detected in New Zealand in August 1997. In the absence of natural predators, rabbits thrive in New Zealand and cause considerable damage to grazing pasture. The covert introduction of this rabbit virus appears to have been motivated by frustration among farmers. Although this biosecurity breach has not had detectable consequences for human health, it illustrates the potential for agents to escape even the most vigilant quarantine systems (7).

### Emergence and Control of Zoonotic Diseases

With the early importation of exotic animals came a limited but important range of zoonotic diseases. These diseases emerged over the past century with the development of an agriculture-based economy. At its peak in the 1980s, New Zealand had 27 production farm animals per capita. Although this figure has fallen to approximately 18 per capita, the occupational hazards for zoonotic disease in New Zealand agricultural workers are higher than in countries where similar diseases occur but the ratio of humans to animals is lower (3). New Zealand has been successful in the control and elimination of some zoonoses; however, others remain problematic.

The threat of introducing plague from the infested ports of post-penal Australia led to the establishment of the New Zealand Department of Health in the late 1800s. Despite improved quarantine, plague did become established in New Zealand as an epizootic of rats in 1900. Human cases occurred from June 1900 until May 1911. Most cases occurred in Auckland, and only one occurred in South Island at the port of Lyttelton. The disease was controlled by a strict port health inspection system, surveillance of arrivals, fumigation of luggage, rat surveillance, and improved building sanitation. Spread was also minimized as the result of low human population density (8).

Anthrax was introduced into New Zealand in the mid-1890s from Calcutta in unsterilized bone dust fertilizer. Outbreaks declined when public health workers, whose infrastructure had been strengthened by the plague effort, imposed sterilization regulations on imported bone dust. The last case of anthrax was recorded in New Zealand in 1954. It is believed that anthrax spores and bacilli are unlikely to persist in New Zealand soils because of high competitive microbial activity (6). As a precaution, several properties remain under active surveillance for the disease.

Historically, *Brucella abortus* was endemic in New Zealand cattle herds and was an important occupational pathogen in farmers and animal slaughterers. A successful animal vaccination and surveillance system resulted in the last indigenous bovine case of brucellosis in New Zealand in 1989; no further indigenous human cases have been recorded (9). *B. suis* and *B. melitensis* are absent from New Zealand, and *Francisella tularensis* is believed to be absent.

Echinococcosis was probably well established in New Zealand before 1873, when it became notifiable. Annual human incidence reached 7 per 100,000 persons between 1900 and 1925. The risk for disease was five to six times higher for New Zealanders of Polynesian descent. Arecoline hydrobromide was introduced in 1908 for treatment of dogs, and an official education program began in 1938. Neither intervention affected the incidence of hydatid disease. In the late 1950s, a massive national effort was undertaken, including the establishment of 800 local voluntary committees throughout New Zealand, education, promotion, peer pressure, dedicated "hydatids officers," and the introduction of the Hydatids Act of Parliament that imposed a levy on dog owners and compulsory dog registration. Canine-stool surveillance and use of arecoline continued until 1972, when it was combined with niclosamide treatment (administered every 6 weeks) and, in 1978, praziquantel. By 1990, active surveillance showed that only three farms were still not free of *Echinococcus granulosus*. In 1999, New Zealand was pronounced provisionally free of hydatids. Human cases of hydatid disease now represent distant past infection, and the New Zealand hydatid control program has been widely regarded as a success (10).

Leptospirosis is endemic in New Zealand and is a frequent cause of disease in farmers and meat workers (11). Of more than 180 recognized serovars of *Leptospira*, only 8 have been isolated in New Zealand. These are *L. interrogans* serovars *australis, canicola, copenhageni*, and *pomona*, and *L. borgpetersenii* serovars *balcanica, hardjobovis, tarassovi,* and *ballum*. Of these, serovars *australis* and *canicola* have been isolated only once each, probably reflecting imported disease in the absence of an endemic animal reservoir. Serovars *hardjobovis, pomona*, and *tarassovi* account for >90% of human leptospirosis cases in New Zealand. Approximately two thirds of disease in dairy farmers is due to serovars *hardjobovis* and one third to serovar *pomona*. Swine farmers are more often infected with serovars *pomona* and *tarassovi*. A readily available animal vaccine, combined with improved milking facilities and sanitation, has contributed to a reduction in human cases. The ongoing annual incidence rate of leptospirosis of 5 per 100,000 persons probably reflects underuse of the animal vaccine. Although serovar *balcanica* is maintained in the possum, transmission of *balcanica* from possums to production animals appears uncommon.

*Mycobacterium bovis* disease is now rare among New Zealanders, partly because of pasteurization of dairy products. However, the elimination of *M. bovis* from cattle herds has proved difficult despite widespread efforts by the veterinary community. New Zealand's maligned Australian brushtailed possum population, estimated at approximately 70 million, emerged as a sylvatic *M. bovis* reservoir in the 1970s and provided a major source of transmission to cattle and deer. Cattle herds in regions of endemic disease are kept under movement control, and a "test and slaughter" policy is in place. The prospect of eliminating animal disease is daunting.

Several zoonotic diseases are notable by their absence. Q fever has been carefully sought for but never found in New Zealand (12). The absence of the disease despite large animal herds, a large farming community, and high levels of animal slaughter is a tribute to careful quarantine, lack of an efficient arthropod vector, and probably an element of good fortune. New Zealand is free of bovine spongiforme encephalopathy because of strict control of animal feed and animal importation. Rabies is also absent from New Zealand, probably reflecting the prolonged duration of the early sea voyage, which exceeded its incubation period in animals; the paucity of indigenous biting animals in the early colonial period; and subsequent strict quarantine practices.

### Endemic and Epidemic Infectious Diseases

The control of infectious diseases in New Zealand during the 1980s and 1990s has been mixed, characterized by persisting and emerging threats, as well as successes and failures (Table).

Table. Key infectious disease threats facing New Zealand

| Infectious disease category | Important examples | Contributing factors |
|---|---|---|
| Vaccine-preventable diseases | Measles<br>Pertussis<br>Influenza | Inadequate vaccine coverage |
| Respiratory-transmitted diseases | Meningococcal disease<br>Tuberculosis<br>Rheumatic fever | Socioeconomic deprivation and crowding |
| Enteric diseases | Campylobacteriosis<br>Salmonellosis<br>Cryptosporidiosis<br>Shiga toxin-producing *Escherichia coli*<br>Yersiniosis<br>Marine biotoxins | High density of reservoir animals<br>Long coastline (in the case of marine biotoxins) |
| Zoonotic disease | Leptospirosis | High levels of infection in animal populations |
| Diseases from contaminated environments | Legionellosis | Uncertain |
| Travel-associated and imported infectious diseases | Hepatitis A<br>Tuberculosis<br>HIV/AIDS | High rates of overseas travel<br>Relatively high levels of immigration |
| Vector-borne diseases | *Ross River virus*<br>*Dengue virus* | Suitable vector mosquitoes and potential NZ habitats |
| Blood-borne diseases | Hepatitis C | Continuing sharing of injecting equipment by injecting drug users |
| Sexually transmitted infections | *Chlamydia*<br>Gonorrhea | Increased levels of sexual activity among young people |
| Hospital-acquired infections | MRSA<br>*Acinetobacter* spp.<br>*Serratia* spp. | Disease burden poorly defined in NZ and no national HAI surveillance system |
| Diseases caused by antibiotic-resistant organisms | Penicillin-nonsusceptible pneumococci<br>VRE<br>MDR-TB | Microbial evolution and incorrect use of antimicrobial agents |

Abbreviations used in this table: NZ, New Zealand; HAI, hospital-acquired infection; MRSA, methicillin-resistant *Staphylococcus aureus*; VRE, vancomycin-resistant enterococci; MDR-TB, multidrug-resistant tuberculosis.

### Infectious Diseases of Poverty and Overcrowding

Certain endemic bacterial diseases have emerged as major causes of illness and death, particularly among New Zealanders of Polynesian descent. These are focused around urban south Auckland and rural parts of North Island, where populations live under difficult socioeconomic conditions.

A serogroup B meningococcal disease epidemic began in 1991 and has persisted for >10 years. Disease rates exceed 12 per 100,000 persons per year, well above those in other industrialized countries (13). Household crowding is an important risk factor, at least for children (14). Identification of the epidemic strain as phenotype B:4:P1.4 provides the opportunity to test the efficacy of a strain-specific serogroup B meningococcal vaccine (15).

After declining for many decades, tuberculosis rates reached a plateau during the 1980s and began to rise during the 1990s. In 1999, 450 cases were reported (12.4 per 100,000), the highest incidence for 20 years (16). Rates among New Zealanders of Polynesian descent are 5 to 15 times higher than among persons of European descent. Immigration from other areas in Oceania (Australia and the Pacific Islands) and high-incidence countries accounts for approximately half the cases each year.

Group A streptococcal disease and its complications are also overrepresented in New Zealanders of Polynesian descent. An average of 100 initial attacks of rheumatic fever have been reported annually in New Zealand over the past 10 years (17). The contributions of environmental and biologic determinants are yet to be established, but there is evidence that both may play a role. Improved management of group A streptococcal disease and secondary prophylaxis for rheumatic fever are research priorities and areas in which New Zealand has special expertise.

Staphylococcal disease is also overrepresented in New Zealanders of Polynesian descent. A recent study of *Staphylococcus aureus* bloodstream infections in Auckland and Christchurch found a rate of community-acquired *S. aureus* bacteremia of 10 per 100,000 per year. Compared with the risk for infection in persons of European descent, the relative risk was 1.8 for indigenous New Zealanders of Polynesian descent (Maori) and 4.0 for nonindigenous New Zealanders of Polynesian descent (18).

### Diseases from Contaminated Environments

*Legionella* infection is an endemic cause of pneumonia, at least in South Island (19), and accounts for 10% of hospital admissions for community-acquired pneumonia. Cases are generally sporadic, and illness is due to various species of *Legionella*.

### Vaccine-Preventable Diseases

New Zealand has a well-developed childhood immunization program, including universal infant hepatitis B immunization and a two-dose measles, mumps, and rubella schedule. However, coverage levels remain low, with barely 60% of children fully immunized by the age of 2 years (20). Consequences include recurring measles (21) and pertussis epidemics (22). Hepatitis B has been overrepresented among those of Polynesian descent (23); hepatitis B surface antigen positivity rates in this population are approximately 5%, compared with 0.5% in New Zealanders of European descent. Annual influenza epidemics, which usually peak around July, also have a large health impact. Free annual vaccination has recently been introduced for those ≥65 years of age and those with chronic medical conditions. However, influenza vaccine coverage remains at only 55% in this age group (24).

### HIV and Other Sexually Transmitted Infections

New Zealand, like Australia, adopted a very progressive response to the arrival of HIV infection and introduced routine control measures early in the epidemic. As a consequence, the prevalence of HIV infection has remained relatively low, particularly among injecting drug users (25). Most cases of HIV/AIDS are in men who have sex with men. The response to other sexually transmitted infections has been far less adequate; consequently, rates of gonorrhea and chlamydia infection appear to be rising (26).

### Enteric Diseases

Enteric infectious diseases rates in New Zealand are among the highest in industrialized countries (27). Culture-confirmed campylobacteriosis occurs at a rate of 305 per 100,000 per year and *Yersinia enterocolitica* at a rate of 14 per 100,000 per year. Furthermore, febrile blood transfusion reactions due to *Y. enterocolitica* occur at the rate of 1 per 65,000 transfusions, reflecting the high number of blood donors with occult bacteremia. These infections occur despite implementation of guidelines to prevent yersiniosis arising from transfused blood (28). Salmonellosis also occurs at relatively high rates in New Zealand. However, the global pandemics of *Salmonella* Enteritidis and *Salmonella* Typhimurium DT104 have not emerged in New Zealand. It is likely that they have been excluded by quarantine practice on animals and animal feed. Giardiasis and cryptosporidiosis (29) rates are also very high. The high rates of endemic enteric infections are not fully understood. The high ratio of domestic production animals to humans and frequent use of rural water supplies in New Zealand have been raised as hypotheses. Perhaps surprisingly, Shiga toxin-producing strains of *Escherichia coli* have emerged relatively recently, but the incidence is now rising rapidly (30). One factor that may contribute to the delayed appearance of this pathogen may be New Zealand's system of pastoral agriculture. Cattle and dairy herds graze year round in open fields, and there is virtually no use of contained feedlots.

New Zealand has a growing aquaculture industry particularly focused on the cultivation of green-lipped mussels (*Perna canaliculus*). As is the case in most temperate regions where commercial shellfish cultivation occurs, nonbacterial forms of seafood poisoning present a potential threat to the industry and human health. A well-developed program of environmental monitoring for dinoflagellate blooms provides an early warning system that prevents potentially toxic shellfish from entering the food chain (31).

### Antimicrobial Resistance

Some global infectious disease threats tend to emerge later in New Zealand than in most other industrialized countries. Delayed occurrence also applies to problems of antimicrobial resistance. One notable exception was the early recognition in 1988 in Auckland of community-acquired methicillin-resistant *Staphylococcus aureus* (MRSA) infection. The relatively susceptible Western Samoan phage patterns predominated (32,33). Multiple drug-resistant MRSA strains remain relatively uncommon, particularly in South Island, where routine screening of staff and patients from areas with endemic MRSA is still an effective hospital infection control practice.

The first vancomycin-resistant *Enterococcus* isolated in New Zealand was reported from Waikato Hospital in 1996. Studies confirm that vancomycin-resistant *E. faecium* and *E. faecalis* are rare (34). The rate of sterile-site isolation of penicillin-nonsusceptible *Streptococcus pneumoniae* was as low as 2.0% in 1993 but rose to 15.1% in 1999. Multiple drug-resistant tuberculosis is very rare and is exclusively imported (35).

### Emerging Infectious Diseases

The disease threats described above are likely to persist unless effective control measures are introduced. In some cases, (e.g., enteric diseases), more research is needed to identify effective prevention and control measures. More often, the measures are well defined but require the political will and resources to introduce them, e.g., to raise immunization coverage, create an environment that encourages safer sexual practices, and address such social determinants as overcrowding. Other disease threats are less predictable and may be caused by the emergence of new organisms, changes of known organisms, and the introduction of previously absent organisms.

### Emergence of New Organisms

To date, human pathogens found in New Zealand have been identified elsewhere. A possible exception is a rickettsial disease first reported from North Island in the 1990s (36,37). Cases were often reported in possum hunters and clustered around the Kaukapakapa region. The rickettsial species, yet to be determined, is probably from the typhus group and may be transmitted to humans from Australian brush-tailed possums by a flea vector. The implication of these animals as a reservoir of an emerging rickettsial disease would only add to the spectra of infectious disease problems and environmental damage that some exotic mammals cause. New Zealand expends considerable resources in pest control.

### Travel and Risk for Imported Diseases

New Zealanders have among the highest per capita international travel rates in the world. Recent data suggest a short-term annual departure rate of 0.31 per capita (38). This compares with 0.22 per capita for Australia and 0.05 per capita for the United States. Oceania and Asia represent the two most popular short-term travel destinations. In addition, New Zealand accepts in excess of 50,000 immigrants annually, predominantly from the same regions. These highly mobile populations provide an ongoing risk for importation of exotic disease. Some of these diseases are capable of becoming established in New Zealand.

### Vector-Borne Disease

No cases of vector-borne disease transmission within New Zealand have been identified. Arboviruses are present, but indigenous circulation of an arbovirus that causes disease in humans has not been documented. The Sindbis-like alphavirus, *Whataroa virus*, is established in bird populations on the west coast of South Island, where human infection without disease has been determined (39,40). The unclassified tick-borne arbovirus, *Johnston Atoll virus*, has been documented in gannet colonies (41).

The potential for introduction of arboviruses currently absent from New Zealand poses an important public health threat (42). The greatest threat is from Australia, a nation with a high incidence of arboviral disease and frequent traveler exchange with New Zealand. Travelers viremic with *Ross River virus* (RRV) and *Dengue virus* arrive with some frequency in New Zealand. Competent exotic vector mosquitoes for both these viruses (i.e., *A. notoscriptus, A. australis*, and *C. quinquefasciatus*) are now established in New Zealand. The efficient RRV vector, *A. camptorhynchus* (the southern salt-marsh mosquito), was also recently introduced from Australia (43). *A. albopictus*, a competent dengue vector, was detected in tires imported from Japan in 1993. This cold-hardy mosquito has not become established in New Zealand but could pose a threat in the future. For climatic reasons, *A. aegypti* is probably precluded from New Zealand, except for the extreme north. *C. annulirostris* from Australia (vector of *Murray Valley encephalitis virus*) and *A. polynesiensis* from the Pacific are vectors of RRV and would be of concern if introduced into New Zealand. Furthermore, a low level of community awareness of and involvement in mosquito control caused by the traditional absence of these diseases would compound the public health effort required to control outbreaks (42).

### Conclusions and Global Relevance

New Zealand provides a unique model for the study of emerging infectious diseases because of its unusual and sparse indigenous fauna, geographic isolation, and relatively recent exposure to humans and exotic animals. Although zoonoses have provided and continue to provide disease control challenges, the economic and structural capacity of New Zealand has allowed several diseases to be eliminated or controlled. Strict agricultural quarantine practices have prevented the reintroduction of these and other zoonotic diseases and helped control human disease. New Zealand's facilities reflect the capacity and limitations of a quarantine system functioning under near optimal circumstances and illustrate the vulnerability of even this system to biocriminal acts. Leptospirosis and bovine tuberculosis provide ongoing challenges.

Group A streptococcal disease, staphylococcal disease, meningococcal disease, and enteric infections are of particular importance in New Zealand; disparities in disease incidence between ethnic groups are of concern. The reason for the high incidence of these infections is being investigated. These and other key communicable disease indicators suggest that further investments in research and control are warranted. A rickettsial disease, probably of the typhus group, is currently emerging in New Zealand. Vectors are established that are capable of transmitting a number of anthropod-borne infections present in neighboring regions and pose a threat should the organisma be imported. Other infectious diseases consistently emerge up to 10 years later in New Zealand than in other industrialized nations. Among these are Shiga toxin-producing strains of *Escherichia coli* and a number of multidrug-resistant bacteria. Here, New Zealand is in the unique position of being able to test recommendations already established in other countries, relatively early in an epidemic.

Although New Zealand invests considerable resources in preventing the introduction of exotic animal and plant diseases, human communicable disease control and research have been inadequately supported. This can be attributed in part to economic constraints and policy but also to complacency that may have arisen from the country's ecologically privileged status for many infectious diseases.

### Acknowledgments

We thank Chuck Landis and Daphne Lee for advice on the geologic development of New Zealand; Jody Scheib for assistance with design of the map; and Helen Heffernan for helpful comments on the manuscript.

Dr. Crump, a New Zealander, is an infectious disease physician and medical microbiologist. He is currently an Epidemic Intelligence Service Officer in the Foodborne and Diarrheal Diseases Branch, Centers for Disease Control and Prevention, Atlanta, Georgia.

### References

1. Sutherland R. Basement geology and tectonic development of the greater New Zealand region: an interpretation from regional magnetic data. Tectonophysics 1999;308:341-62.
2. Pool DI. The effects of the 1918 pandemic of influenza on the Maori population of New Zealand. Bull Hist Med 1973;47:273-81.
3. Wilks C. Zoonoses in New Zealand. 2nd ed. Palmerston North: Foundation of Continuing Medical Education of the New Zealand Veterinary Association; 1997.
4. Poole A. Wild animals of New Zealand. 1st ed. Wellington, New Zealand: A.H. & A.W. Reed; 1970.
5. Tenquist J, Charleston W. An annotated checklist of ectoparasites of terrestrial mammals in New Zealand. Journal of the Royal Society of New Zealand 1981;11:257-85.
6. Hughes KL. History of veterinary public health in Australasia. Rev Sci Tech 1991;10:1019-40.
7. Greenslade E, Weinstein P, Woodward A, Capucci L, Salmond C, Beasley R. A serological survey of antibodies to rabbit haemorrhagic disease virus (rabbit calicivirus disease) in two rural Central Otago communities. N Z Med J 2001;114:55-8.
8. MacLean FS. The history of plague in New Zealand. N Z Med J 1955;54:131-43.
9. Chereshsky A, Wright J, Baker M. Human brucellosis: no evidence of transmission in New Zealand. New Zealand Public Health Report 1997;4:3-5.
10. Burridge MJ, Schwabe CW, Fraser J. Hydatid disease in New Zealand: changing patterns in human infection, 1878-1972. N Z Med J 1977;85:173-7.
11. Thornley C, Baker M, Mass M. Descriptive epidemiology of leptospirosis in New Zealand, 1990 through 1998. Marysville, Australia: International Leptospirosis Society; 1999.
12. Hilbink F, Penrose M, Kovacova E, Kazar J. Q fever is absent from New Zealand. Int J Epidemiol 1993;22:945-9.
13. Baker M, Martin D, Kieft C. The evolving meningococcal disease epidemic in New Zealand. New Zealand Public Health Report 1999;7:57-61.
14. Baker M, McNicholas A, Garrett N, Jones N, Stewart J, Koberstein V, et al. Household crowding a major risk factor for meningococcal disease in Auckland children. Pediatr Infect Dis J 2000;19:983-90.
15. Martin DR, Walker SJ, Baker MG, Lennon DR. New Zealand epidemic of meningococcal disease identified by a strain with phenotype B:4:P1.4. J Infect Dis 1998;177:497-500.
16. Tuberculosis in 1999: highest number of cases since 1980. New Zealand Public Health Report 2001;8:4-5.
17. Naing T, Baker M, Martin D, Crampton P. New Zealand trends in rheumatic fever and chronic rheumatic heart disease 1980-1998. In: Martin DR, Tagg JR, editors. Streptococci and streptococcal diseases: entering the New Millennium. Proceedings of the XIV Lancefield International Symposium on Streptococci and Streptococcal Disease, Auckland, New Zealand, 1999. New Zealand: Securacopy; 2000. p. 537-9.
18. Hill P, Birch M, Chambers S, Drinkovic D, Ellis-Pegler RB, Everts R, et al. Prospective study of 424 cases of *Staphylococcus aureus* bacteremia: determination of factors affecting incidence and mortality. Intern Med J 2001;31:97-103.
19. Chambers ST, Town GI, Neill AM, Frampton C, Murdoch DR. *Legionella*, *Chlamydia pneumoniae* and *Mycoplasma* infection in patients admitted to Christchurch Hospital with pneumonia. N Z Med J 1999;112:222-4.
20. Rainger W, Soloman N, Jones N. Immunisation coverage and risk factors for immunisation failure in Auckland and Northland. New Zealand Public Health Report 1998;5:49-52.
21. Mansoor O, Blakely T, Baker M, Tobias M, Bloomfield A. A measles epidemic controlled by immunisation. N Z Med J 1998;111:467-71.
22. Blakely T, Mansoor O, Baker M. The 1996 pertussis epidemic in New Zealand: descriptive epidemiology. N Z Med J 1999;112:30-3.
23. Blakely T, Salmond C, Tobias M. Hepatitis B virus carrier prevalence in New Zealand: population estimates using the 1987 police and customs personnel survey. N Z Med J 1998;111:142-4.
24. Jennings L, Huang S, Baker M, Bonne M, Galloway Y, Baker S. Influenza surveillance and immunisation in New Zealand, 1990-1999. New Zealand Public Health Report 2001;8:9-12.
25. Paul C, Wilson M, Dickson N, Sharples K, Skegg D. Enhanced surveillance of HIV infections in New Zealand. N Z Med J 2000;113:390-4.
26. Turley M, McNicholas A, Nesdale A, Bennett S, Garrett N. Sexually transmitted infections at New Zealand sexual health clinics. New Zealand Public Health Report 2000;7:49-52.
27. Lake R, Baker M, Garrett N, Scott W, Scott H. Estimated number of cases of foodborne infectious disease in New Zealand. N Z Med J 2000;113:278-81.
28. Theakston EP, Morris AJ, Streat SJ, Baker BW, Woodfield DG. Transfusion transmitted *Yersinia enterocolitica* infection in New Zealand. Aust N Z J Med 1997;27:62-7.
29. Duncanson M, Russell N, Weinstein P, Baker M, Woodward A, Skelly C. Regional rates of notified cryptosporidiosis compared with drinking-water supply quality in Aotearoa New Zealand. Journal of Water Research 2000;34:3804-12.
30. Baker M, Eyles R, Bennett J. Emergence of verotoxigenic *Escherichia coli* (VTEC) in New Zealand. New Zealand Public Health Report 1999;7:9-12.
31. Sim J, Wilson N. Surveillance of marine biotoxins. N Z Public Health Report 1997;4:9-11.
32. Mitchell JM, MacCulloch D, Morris AJ. MRSA in the community. N Z Med J 1996;109:411.
33. Riley D, MacCulloch D, Morris AJ. Methicillin-resistant *S. aureus* in the suburbs. N Z Med J 1998;111:59.
34. Kobayashi K, Rao M, Keis S, Rainey FA, Smith JMB, Cook GM. Entrococci with reduced susceptibility to vancomycin in New Zealand. J Antimicrob Chemother 2000;46:405-10.
35. Brett M, Ellis-Pegler R. Surveillance of antimicrobial resistance in New Zealand. New Zealand Public Health Report 2001;8:17-21.
36. Ellis-Pegler RB, Cooper IP, Croxson MC. Murine typhus in Kaukapakapa? N Z Med J 1991;104:333-4.
37. Roberts SA, Ellis-Pegler RB. Murine typhus in the Kaukapakapa area again. Aust N Z J Med 1997;27:446-7.
38. Statistics New Zealand/Te Tari Tatau. Profile of New Zealand, 2000. Available at: http://www.stats.govt.nz/. Accessed August 2001.
39. Maguire T, Miles JA, Casals J. Whataroa virus, a group A arbovirus isolated in South Westland, New Zealand. Am J Trop Med Hyg 1967;16:371-3.
40. Miles JA. The ecology of Whataroa virus, an alphavirus, in South Westland, New Zealand. J Hyg 1973;71:701-13.
41. Austin FJ. Johnston Atoll virus (Quaranfil group) from *Ornithodoros capensis* (*Ixodoidea: Argasidae*) infesting a gannet colony in New Zealand. Am J Trop Med Hyg 1978;27:1045-8.
42. Weinstein P, Laird M, Calder L. Australian arboviruses: at what risk New Zealand? Aust N Z J Med 1995;25:666-9.
43. Hearnden M, Skelly C, Dowler H, Weinstein P. Improving the surveillance of mosquitoes with disease-vector potential in New Zealand. New Zealand Public Health Report 1999;6:25-8.
